# Validate the force-velocity relation of the Hill’s muscle model from a molecular perspective

**DOI:** 10.3389/fbioe.2022.1006571

**Published:** 2022-10-14

**Authors:** Yongkun Zhao, Shihang Ding, Masahiro Todoh

**Affiliations:** ^1^ Division of Human Mechanical Systems and Design, Graduate School of Engineering, Hokkaido University, Sapporo, Japan; ^2^ Division of Bioengineering, Graduate School of Engineering Science, Osaka University, Osaka, Japan; ^3^ Division of Mechanical and Aerospace Engineering, Faculty of Engineering, Hokkaido University, Sapporo, Japan

**Keywords:** neuromechanics, skeletal muscle, Hill’s muscle model, parallel cluster model, force-velocity relation

## 1 Introduction

Today, most countries have become ageing societies (Lee et al., 2020). Motion disorder is one of the most common age-related diseases which is worth investigating. Several researchers are studying methods to model muscles in order to simulate human motions since muscles are the engines of human motion (Mathis and Schneider, 2021; Takei et al., 2021; Ueyama, 2021). The Hill’s muscle model is most commonly used for addressing this issue in a great deal of research. Most researchers dismiss its force-velocity relation as a phenomenological paradigm (Miller, 2018). However, it is extremely important to validate the applicability of this relation obtained from the model. Therefore, this paper aims to provide an explanation to support that from a molecular perspective. In detail, this work is broken into three sections, the first of which describes the mechanism of muscle contraction, the second of which describes the intricacies of the Hill-based muscle model, and the third of which connects the first two sections and tries to give molecular validation for the force-velocity relation of the Hill’s muscle model.

## 2 Muscle activities from a molecular aspect

Skeletal muscle, a form of striated muscle, is extremely important in the human body. Its structure and contraction mechanisms have been elucidated (Huxley, 1957; Caremani et al., 2021; Dowling et al., 2021). A description of the entire process of muscle contraction, including the signaling system and molecular interactions, is given here in order to provide insights into the explanation of Hill function from a microscopic perspective.

### 2.1 Electrochemical signaling

Skeletal muscle is made up of multiple muscle fibers, which can termed as skeletal muscle cells as well (Frontera and Ochala, 2015). These muscle fibers facilitate the force propagation from them to the tendon due to their highly ordered arrangement. In each muscle fiber, myofibrils which present the filamentous structure are parallel to each other, and they are covered by the sarcolemma. In other words, the sarcolemma is the membrane of the muscle cell. On this membrane, there exists a region called the motor end-plate on which neurotransmitter receptors are extensively distributed (Katz and Thesleff, 1957; Kuo and Ehrlich, 2015). Based on this, neuromuscular junctions can be formed when motor neurons branch into several presynaptic terminals near the motor end-plate and combine with the receptors. For activating the muscle contraction, the somatic nervous system (SNS), a significant part of the peripheral nervous system (PNS), will generate the neural impulse as an electric signal that can turn on the voltage-gated calcium channels on each synapse (Mai and Paxinos, 2011). As a result, calcium ions enter those synapes and consequently trigger the release of acetylcholine (ACh), which is a kind of neurotransmitter and working as a chemical signal.

### 2.2 Release of the cytosolic calcium

**FIGURE 1:**
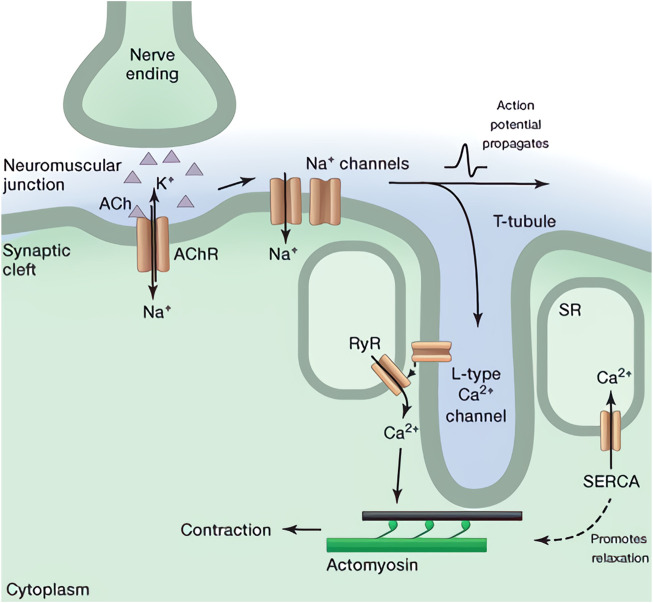
Illustration of force generation in skeletal muscle including signaling pathway and DICR (Kuo and Ehrlich, 2015).

On the sarcolemma, nicotinic acetylcholine receptors (nAChRs) can bind to the ACh when it diffuses through the synaptic cleft (Karlin, 2002). As a result, the sodium channel that is a ligand-gated iontropic receptor can be mechanically opened by the activated nAChRs, since they are directly connected to each other. Therefore, the subsequent influx of sodium ions triggers the sarcolemma depolarization and the formation of the action potential (Bean, 2007). This potential will travel along the membrane until it reaches the transverse tubules, which are groove structures on the sarcolemma that contain a significant number of ion channels. In these transverse tubules, a huge number of L-type calcium channels that control the excitation-contraction coupling in skeletal muscle will be activated by the action potential (Kuo and Ehrlich, 2015). And their conformational shift will mechanically drag the ryanodine receptors (RyRs) in the sarcoplasmic reticulum (SR). Considering the RyR, it is divided into three isoforms (RyR1, RyR2, and RyR3) that correspond to the calcium release in cells (Fill and Copello, 2002; Gong et al., 2021; Woll and Van Petegem, 2022). It should be noticed that the RyR1 is the receptor which is found predominantly in skeletal muscle and is linked to the L-type calcium channel directly. This RyR1 increases the release of calcium stored in SR after being activated *via* the L-type calcium channel, resulting in a rise in cytosolic calcium concentration. Depolarization-induced calcium release (DICR) is another term for this phenomenon. Illustration of this process can be found in Figure 1.

### 2.3 The crossbridge model and contraction

**FIGURE 2:**
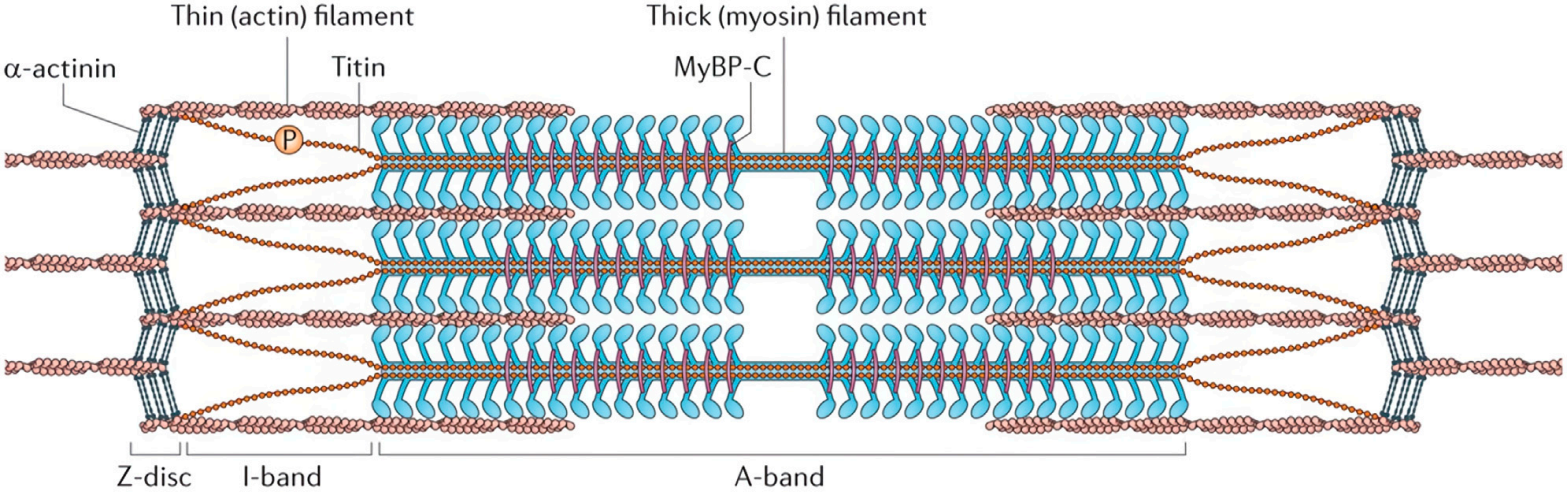
Illustration of the sarcomere structure (Hwang and Sykes, 2015). The I-band corresponds to the region of F-actins observed by microscope, and the A-band represents the overlapping area of the myosin thick filament and the F-actins.

Considering in each myofibril, periodic patterns along its longitudinal direction can be observed clearly (Huxley, 1957; Herzog, 2017, 2022). Such a repeating structure is termed as the sarcomere, which is a highly organized architecture predominantly composed of multiple filamentous actins (F-actins), myosin thick filaments, titins, and Z-disc proteins (*α*-Ctinins) as shown in Figure 2 (Hwang and Sykes, 2015). For a single F-actin, it is composed of multiple golobular actin proteins (G-actins). Due to the inherently asymmetrical structure of G-actins and their regular orientation, each F-actin exhibits a spatial polarity with the barbed and the pointed ends (Carlsson, 2010; Dominguez and Holmes, 2011). In consideration of the sarcomere, however, a symmetrical structure can be observed. Such a spatial symmetry is formed by two sets of bundled F-actins with opposite polarity which are connecting to distinct Z-discs. In both sets of them, their pointed ends are left free, while the barbed ends are fastened on the Z-discs. According to the previous experimental works and the crossbridge model, myosin-II proteins tend to move toward the barbed ends of F-actins during their interactions. It should be noticed that the myosin-II is the non-processive protein since it can easily dissociate from the substrate. Therefore, multiple myosin-II proteins usually associate together through their tail domains as a bipolar structure called myosin thick filament to achieve the processive movement (Sweeney and Houdusse, 2010). Due to the symmetry structure, this thick filament can connect to the two sets of F-actins and locates at the core of each sarcomere due to titins and the myosin-binding protein C (MyBP-C) (Tskhovrebova and Trinick, 2003; Hwang and Sykes, 2015). Meanwhile, multiple myosin heads exist in both sides of the thick filament which perform a stochastic property according to the crossbridge model (Huxley, 1957).

As the cytosolic calcium concentration rises, the released calcium ions can bind to the protein troponin C on F-actin, which is a component of the troponin complex (Galińska-Rakoczy et al., 2008). Then, a conformational change of this complex will mechanically shift the tropomyosin to the groove of the helical F-actin (Galińska-Rakoczy et al., 2008; Von der Ecken et al., 2015). Consequently, the myosin-binding sites on F-actins can be exposed, thus resulting in the sustain movement of myosin thick filament toward the barbed ends. During this process, F-actins cross the thick filament and shorten the sarcomere length which leads to the muscle contraction eventually. Considering the molecular connection between the myosin-II protein and F-actin, it should be researched further to better comprehend the muscle contraction. Advances in single-molecule experiments have revealed precise knowledge about myosin architectures (Ferrer et al., 2008; Sweeney and Houdusse, 2010; Yang et al., 2020; Rahmani et al., 2021). Based on these investigations, myosin-II can be mainly classified into three domains: the head domain, the lever arm domain, and the tail domain. The heads of myosin-II in skeletal muscle can associate with the F-actin, ATP, and its hydrolysis products. Meanwhile, the angle between the head and the coiled-coil tail can be adjusted by the lever arm domain. It also serves as a binding site for the myosin light chains (MLCs) that contribute to the myosin integrity and modulating the ATP hydrolysis. In addition, the coiled-coil part is an elastic structure that resembles a tail in myosin. It can form the myosin thick filament by combining with that of other myosin-II proteins.

According to the crossbridge model (Huxley, 1957), the cyclical interactions between myosin proteins and F-actins are the origins of contractile force. Among these interactions, a local conformational change of the myosin is essential for the force generation, which is described as a “swinging” in the crossbridge model. Based on this, myosin-II can make a step toward the barbed ends of F-actins. To describe this process, mainly two scenarios have been proposed: the power stroke and the Brownian ratchet (Bustamante et al., 2001; Holmes et al., 2004; Moretto et al., 2022). The mechanism of power stroke illustrates how a conformational shift in myosin occurs, causing the myosin to take an irreversible step toward the barbed end. Meanwhile, the Brownian ratchet mechanism states that the arm of myosin would randomly reach forward and backward binding-sites due to its thermal fluctuation, and that the forward binding-site becomes the preferential one as a result of energy events and myosin conformational change. However, according to a recent comparison, these two cases are not conceptually dissimilar. The Brownian ratchet may be a better explanation for a reduced step size. Myosin dynamics, on the other hand, can be considered as power strokes when the ratchet-like motion increments are tiny enough in comparison to the step size (Hwang and Karplus, 2019; Matusovsky et al., 2020; Marcucci et al., 2021). As a result, the myosin dynamics are referred to as a power stroke mechanism for a more intuitive comprehension.

**FIGURE 3:**
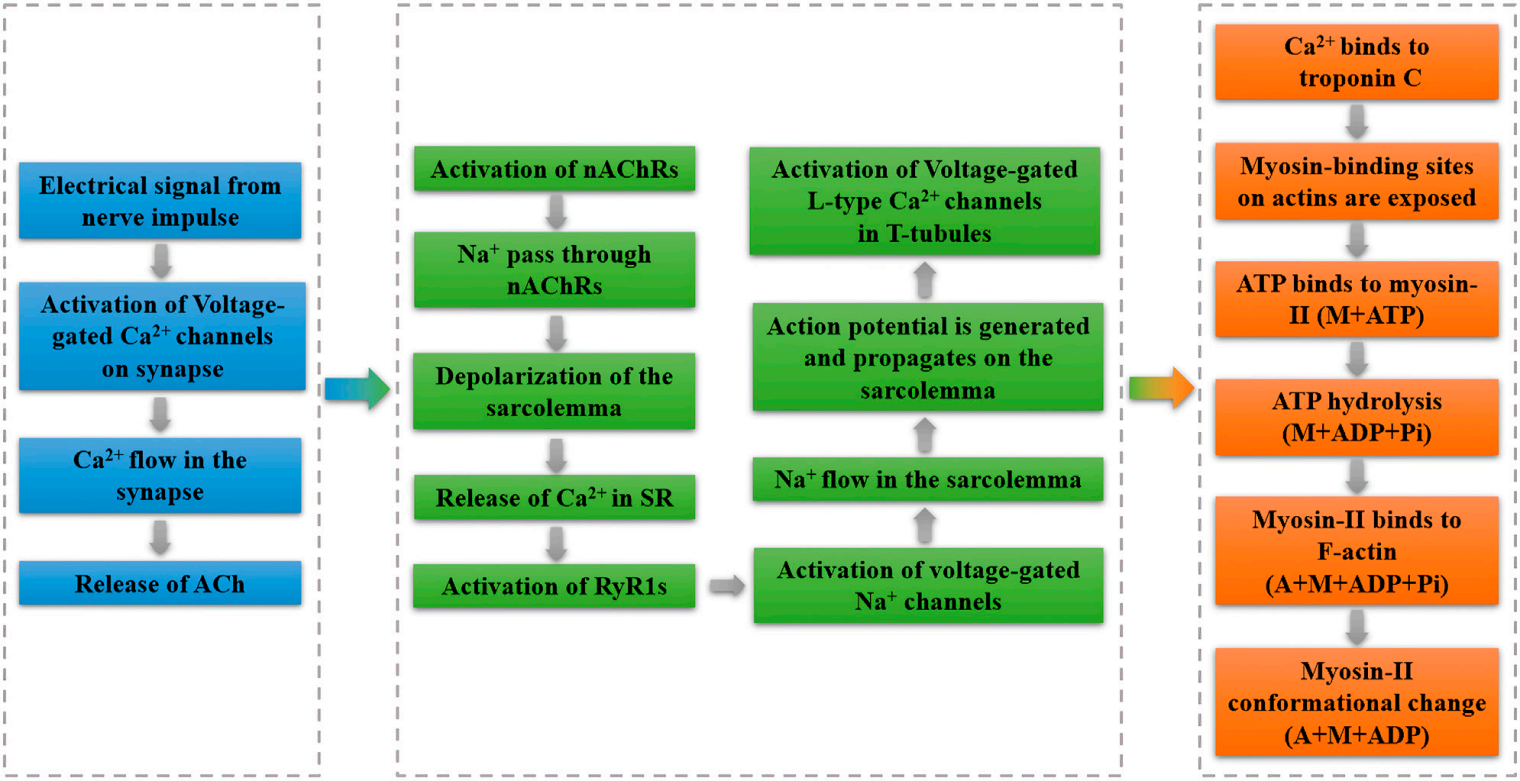
Process of muscle contraction. Part in blue represents the electrochemical signaling; part in green denotes the process of DICR; part in orange indicates the crossbridge model of actomyosin interactions.

Based on the crossbridge model and the mechanism of power stroke (Huxley, 1957; Hwang and Karplus, 2019), the entire process of the actomyosin interactions can be described in detail. After the shift of tropomyosin, myosin-II heads can bind to the F-actins by consuming ATP. During this process, the hydrolysis products (ADP and phosphate) are still attaching on the myosin head. Following that, the phosphate (Pi) release triggers a conformational shift in the lever arm, which pulls the F-actin and causes a contraction. After releasing the ADP, myosin head can detach from and reattach to the F-actin by associating with a new ATP and hydrolysing it. Within these cyclical interactions, myosin can continuously bind to a new binding site because a relative displacement has already happened. The overall ensemble of myosin can constantly travel to the barbed end by repeating such contacts, resulting in skeletal muscle processive contraction. The complete process of muscle contraction is illustrated as Figure 3.

## 3 Hill-based muscle modeling

**FIGURE 4:**
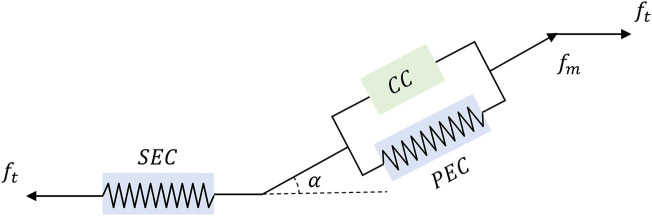
A diagram of Hill-based muscle model includes CC, PEC, and SEC, where *f*_*t*_ and *f*_*m*_ represents the muscle tendon force and muscle fiber force. And *α* is the pennation angle, which is defined as the angle between the direction of muscle fibers and the line of action of the muscle force.

The Hill-based muscle model is the most widely used computer model for human movement modeling (Miller, 2018). The Hill-model is made up of three parts: contractile, series elastic, and parallel elastic components. A contractile component (CC) is in series with a series elastic component (SEC), and both of them are parallel to the parallel elastic component (PEC) in a Hill-based muscle model (Scovil and Ronsky, 2006). To visualize such a process, the illustration is depicted in Figure 4. Parameters in this section can be found in Table 1.

**TABLE 1:** Parameters in the Hill muscle model.

Parameter	Symbol	Unit
Maximum isometric force	*f*_0_	[m]
Force-length relation	*f*_*fl*_	None
Force-velocity relation	*f*_*fv*_	None
Activation level	*a*	None
Pennation angle	*α*	[°]
Muscle tendon force	*f*_*t*_	[N]
Muscle fiber force	*f*_*m*_	[N]
Contractile component force	*f*_*cc*_	[N]
Series elastic component force	*f*_sec_	[N]
Parallel elastic component force	*f*_*pec*_	[N]
Length of series elastic component	*l*_sec_	[m]
Length of parallel elastic component	*l*_*pec*_	[m]
Unloaded length	*l*_*u*_	[m]

### 3.1 Contractile component

A neural signal is generated and delivered to the muscular system after a motor neuron is excited. This signal is classified as a time-varying signal under the Hill model, and it refers to the sum of the motor unit action potentials. And the signal’s value is configured to be between 0 and 1. The CC activates after receiving this signal, and then transfers this signal to the activation level, which similarly has a value range of 0–1. It should be noted that this activation takes time to complete, as this corresponds to the time required for a chemical reaction. At the sarcomere level, the actin and myosin cross-bridges are the source of force created by CC. The Hill model assumes that the value of the CC force produced is determined by three factors: the force-length relationship, the force-velocity relationship, and the activation level mentioned earlier. It should be noted that these three factors are commonly assumed as multiplicative and independent of others (Miller, 2018). The following is the formula for it (Miller, 2018):

$$f_{cc}=f_{0}\cdot a\cdot f_{fl}\cdot f_{fv}$$ (1)

where, *f*_0_ represents the maximum isometric force, *f*_*fl*_ and *f*_*fv*_ represent the relationship of force-length, and force-velocity, and both of them are nondimensional and *a* is the activation level.

### 3.2 Series elastic component

The SEC follows an elastic force-extension relationship. It should be noted, however, that the SEC is neglected for several tendon types due to its high stiffness (Winters, 1990). When the length of SEC is less than the unloaded length, the force created by SEC is typically regarded to be zero. When the length of the SEC is increased over the unloaded length during a contraction, the SEC force generates progressively more force. A nonlinear model was built to capture the force-extension relationship in order to quantitatively express this phenomenon (Pawlak, 2022).

$$\begin{array}{cc} f_{sec} & =\left\{\begin{array}{cc} \gamma \left(l_{sec}-l_{u}\right)\; & \text{if }l_{sec}>l_{u}\\ 0\; & \text{if }l_{sec}\leq l_{u} \end{array}\right.\\ \gamma & =f_{0}/{\left(u_{0}l_{u}\right)}^{2} \end{array}$$ (2)

where, *l*_sec_ and *l*_*u*_ are lengths of SEC and its unloaded length. *u*_0_ is a constant factor which is commonly set as 0.04, and the value of it significantly affects the muscle energetics (Lichtwark and Wilson, 2007).

### 3.3 Parallel elastic component

Aside from the CC and SEC, PEC is another component in the Hill muscle model which has a force-extension relationship that is parallel to that of the CC only. However, sometimes PEC can be also set parallel to the both CC and SEC (Lo and Xie, 2012). The force generated by PEC is modeled as a function of both *f*_*cc*_ and *f*_sec_ (Blemker, 2017).

$$f_{\mathrm{pec}}\cos\alpha =f_{sec}-f_{cc}\cos\alpha$$ (3)

$$f_{t}=f_{\mathrm{pec}}+f_{sec}=f_{\mathrm{pec}}+f_{cc}\cos\alpha$$ (4)

where *f*_*t*_ denotes the total force exerting on the muscle tendon.

## 4 Molecular validation for the force-velocity relation

In Section 2, the entire muscle contraction process including the signaling system and molecular interactions is described. To validate the force-velocity relation of the Hill-based muscle model quantitatively, a precise mathematical model describing the myosin activities have to be constructed. Hence, in this section we introduce a model of myosin ensemble called a “Parallel Cluster Model” and present numerical simulation results on it. Its force-velocity diagram exhibits a similar shape as that of the Hill-based muscle model.

### 4.1 Parallel cluster model

In order to quantitatively validate the Hill model, the parallel cluster model (PCM) is adopted which can replicate the force-velocity relationship of a myosin ensemble (Erdmann et al., 2013). Parameters in this PCM can be found in Table 2. According to the mechanism of power stroke, myosin proteins can be classified into three states. Assume that an ensemble has *N* number of myosin-II proteins, with number *i* of them binding to F-actins. As a result, the number of free state quantity of myosin is *N* − *i*. The number *j* of the bound myosin proteins are in the post-power-stroke state (after conformational change of the lever arm). As a result, number *i* − *j* myosin molecules are in the pre-power-stroke condition. The free, pre-power-stroke, and post-power-stroke states are represented by the subscripts 0, 1 and 2, respectively. Myosin’s coiled-coil domain can be simplified into a spring-like structure with a stiffness of *k*_*m*_ (Vilfan and Duke, 2003). Basically, other myosin groups will exert force on each ensemble due to the symmetry structure of the myosin thick filament. Based on the assumption that the myosin monomers in the same state are bearing equivalent load (Erdmann and Schwarz, 2012; Erdmann et al., 2013), the elastic force in myosin ensemble can balance such an external force, as shown below:

$$f=k_{m}\left[\left(i-j\right)\varepsilon_{ij}+j\left(\varepsilon_{ij}+d\right)\right]=k_{m}\left(i\varepsilon_{ij}+jd\right)$$ (5)

where *ɛ*_*ij*_ stands for the myosin strain in condition (*i*, *j*). The increased strain induced by myosin conformational change is indicated by *d* (Vilfan and Duke, 2003). When the external force is a constant value, the force generated by the myosin group is independent of the ensemble position (Erdmann et al., 2013). As a result, the strain *ɛ*_*ij*_ can be expressed as follows:

$$\varepsilon_{ij}=\frac{1}{i}\left[\left(f/k_{m}\right)-jd\right]$$ (6)

**TABLE 2:** Parameters in the parallel cluster model.

Parameter	Symbol	Unit
Total number of myosin	*N*	None
Number of bound myosin	*i*	None
Number of post-power-stroke myosin	*j*	None
Stiffness of myosin neck linker	*k*_*m*_	[pN/nm]
External force	*f*	[pN]
Strain of pre-power-stroke myosin	*ϵ*_*ij*_	[nm]
Strain of power-stroke	*d*	[nm]
Transition rates	*k*_01_, *k*_10_, *k*_12_, *k*_21_, *k*_20_	[s^−1^]
Zero-force unbinding rate	$k_{20}^{0}$	[s^−1^]
Elastic force on post-power-stroke myosin	$f_{ij}^{pp}$	[pN]
Total energy of bound myosin	*e*_*ij*_	[pN nm]
Thermal energy	*k*_*B*_ *T*	[pN nm]
Energy bias to post-power-stroke state	*e*_*pp*_	[pN nm]
Position change of ensemble caused by new binding	Δ*Z*_*on*_	[nm]
Unbinding force	*f*_0_	[pN]
Ensemble velocity in condition *i*	*V*_*i*_	[nm/s]
Averaged velocity of bound ensemble	*V*_*b*_	[nm/s]
Unloaded velocity of muscle	*v*_0_	[nm/s]
Stall force of myosin	*f*_*s*_	[pN]
Velocity derived from the Hill muscle model	*v*_*hill*_	[nm/s]

With transition rates of Tr_01_, Tr_10_, Tr_12_, Tr_21_, and Tr_20_, transitions between myosin states can be characterized in a stochastic fashion. Only the transition from state 2 to state 0 is irreversible since the release of ADP and hydrolysis of a new ATP occur during the unbinding events. Except for Tr_20_, all transition rates in the PCM can be thought of as constants derived from single molecule studies (Vilfan and Duke, 2003; Walcott et al., 2012). The unbinding rate Tr_20_ for myosin heads in state 2 can be stated as follows, according to the reaction-rate hypothesis (Hänggi et al., 1990).

$${Tr}_{20}\left(i,j\right)={Tr}_{20}^{0}exp\left(-f_{ij}^{pp}/f_{0}\right)$$ (7)

where ${Tr}_{20}^{0}$ represents the constant zero-force unbinding rate as determined by experimental studies. The elastic force acting on individual myosin in the post-power-stroke condition is represented by $f_{ij}^{pp}=k_{m}\left(\varepsilon_{ij}+d\right)$ . The force triggered by the unbinding event is indicated by the constant *f*_0_, which is a theoretical force equals to the quotient of the local thermal energy divided by unbinding distance (Vilfan and Duke, 2003; Walcott et al., 2012).

This expression of the unbinding rate fits the catch-bond property of myosin-II well. When a binding myosin is subjected to a tensile force, a catch-bond occurs, in which the bond becomes stronger or the dwell duration of the binding molecule increases (Thomas et al., 2008; Barger et al., 2020; Xie, 2020). This catch-bond trait can be described using a variety of conceptual and quantitative models. The ligand and receptor combine like hooks in the conceptual description. The tensile force can strengthen the connection due to its rigidity. In terms of quantitative models, the majority of them follow the reaction-rate theory described above, which explains the unbinding event from the perspective of interaction potential and force (Pereverzev et al., 2005; Cortes et al., 2020; Weirich et al., 2021). According to these explanations, if there is an external force acting on the protein, myosin attachment will be firmed. And such a response can result in a concave force-velocity curve, which can also be deduced from the Hill muscle model.

In addition, a local thermodynamic equilibrium in each myosin ensemble can be assumed, since the state transitions occur in the time scale of nanoseconds while the muscle activities occurs in milliseconds (Jülicher et al., 1997; Moretto et al., 2022). Moreover, the length scale of myosin movements is only in nanometers which is extremely short compared to the cellular activities. These comparison of order of magnitude indicate the local thermodynamic equilibrium for myosin dynamics is necessary. Therefore, the Boltzmann distribution can be used to compute the conditional probability to find *j* myosin proteins in the post-power-stroke state and *i* − *j* in the pre-power-stroke state for a given number *i* of bound myosin.

$$Pr\left(j|i\right)=\frac{1}{Q_{i}}exp\left(-e_{ij}/k_{B}T\right)$$ (8)

$$Q_{i}=\overset{i}{\underset{j=0}{\sum }}exp\left(-e_{ij}/k_{B}T\right)$$ (9)

where *Q*_*i*_ is the partition sum. *e*_*ij*_ represents the total energy of bound myosin. *k*_*B*_ indicates the Boltzmann constant, *T* denotes the temperature.

The release of phosphate, elastic energy in myosin, and the contribution of external force are the three main sources of energy in bound myosin. External contribution can be ignored because *f* is unaffected by myosin position. As a result, *e*_*ij*_ can be computed as follows:

$$e_{ij}=je_{pp}+\frac{k_{m}}{2}\left[\left(i-j\right)\varepsilon_{ij}^{2}+j{\left(\varepsilon_{ij}+d\right)}^{2}\right]$$ (10)

where *e*_*pp*_ denotes the energy bias from pre-power-stroke state to post-power-stroke state triggered by phosphate release (Vilfan and Duke, 2003).

Myosin’s lever arm moves forward by *d* triggered by the power stroke. The elastic domain in myosin is stretched during this conformational change. This transition, however, does not change the position of the bound myosin head. All bound myosin heads are assumed to be in the same position in the parallel cluster model. Additionally, the ensemble position is defined as the average of the bound myosin heads (Erdmann and Schwarz, 2012; Erdmann et al., 2013). Therefore, the transition from 1 to 2 and most unbinding events would not change the ensemble position. However, the backbone of the myosin thick filament moves forward due to the bending of the lever arm. Thus, the new binding of a free myosin (from 0 to 1) occurs in a different location and pulls the entire ensemble accordingly. Furthermore, in the situation where there is only one myosin bound to F-actin, the unbinding of this myosin will alter the position of the ensemble. In the meantime, this unbinding will move the ensemble by a distance equal to the strain value of the myosin. The following formula can be used to compute the position change of the ensemble caused by a new binding (Erdmann et al., 2013):

$$\Delta Z_{on}=-\frac{1}{i+1}\overset{i}{\underset{j=0}{\sum }}\varepsilon_{ij}Pr\left(j|i\right)$$ (11)

As a result, the velocity of a myosin ensemble can be calculated by multiplying the binding or unbinding rate by its change of ensemble position (Erdmann and Schwarz, 2012; Erdmann et al., 2013). The following equation is how velocity can be calculated using this definition:

$$V_{i}=\left(N-i\right){Tr}_{01}\Delta Z_{on}-\left[{Tr}_{10}\varepsilon_{10}Pr\left(j=0|i=1\right)+{Tr}_{20}\left(1,1\right)\varepsilon_{11}Pr\left(j=1|i=1\right)\right]\delta_{i1}$$ (12)

where *V*_*i*_ is the ensemble velocity with condition *i* and *f* is the force contained in the myosin strain *ɛ*_*ij*_. The contribution of binding events is represented by the first term, while the contribution of the latest unbinding event is represented by the second term. *δ*_*i*1_ is the Kronecker delta, which can be used to determine whether the only bound myosin is still present.

Considering a general situation, *ɛ*_*ij*_, Tr_20_ and Pr (*j*|*i*) need to be updated with each change of *i*. With the Gillespie method (Gillespie, 1976) based on standard Monte-Carlo inversion steps, the averaged velocity of bound ensemble (*i* ⩾ 1) in the parallel cluster model (Erdmann et al., 2013) can be expressed as follow:

$$V_{b}=\overset{N}{\underset{i=1}{\sum }}V_{i}{\hat{Pr}}_{i}\left(\infty \right)$$ (13)

where ${\hat{Pr}}_{i}\left(\infty \right)$ indicates the re-normalized distribution of the stationary probability to find a bound ensemble (*i* ⩾ 1) over infinite time (Erdmann and Schwarz, 2012; Erdmann et al., 2013).

**FIGURE 5:**
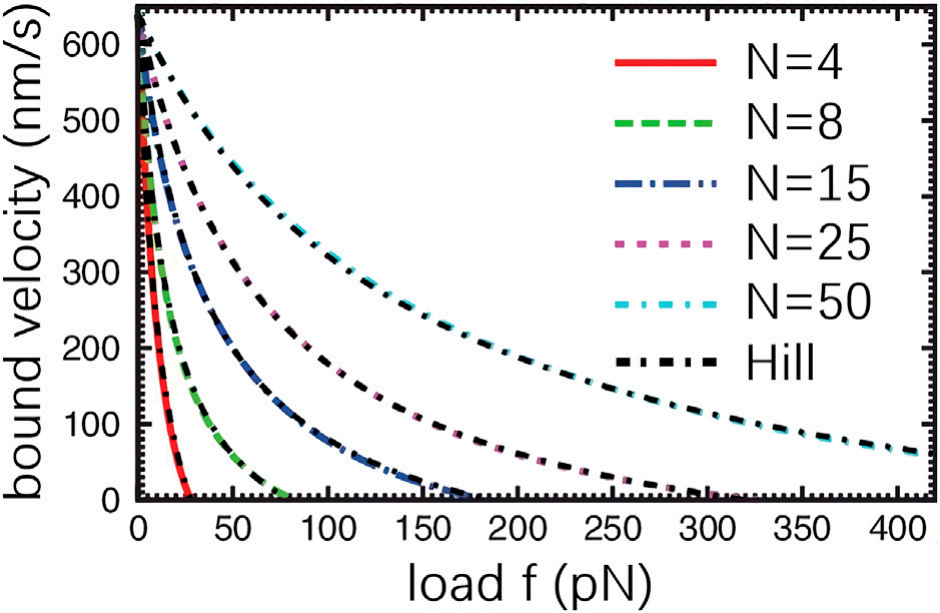
Force-Velocity relationship derived from PCM (Erdmann et al., 2013). *f* indicates the external force acting on the myosin ensemble. *N* represents the total number of myosin in the ensemble. The black, dashed curve denotes the result obtained from the Hill muscle model.

For the comparison, numerical simulation experiments on both Hill-based muscle model and parallel cluster model are conducted. As can be seen from Figure 5, the force-velocity diagram generated by the Hill muscle model fits the force-velocity diagram generated by PCM very well. From the simulation results obtained from the literature (Erdmann et al., 2013) demonstrate a qualitative agreement between the Hill model and the force-velocity curve of muscle simulation by PCM.

The relationship between force and velocity derived from the Hill muscle model is shown as follows (Erdmann et al., 2013; Cortes et al., 2020).

$$v_{\mathrm{hill}}=v_{0}\frac{f_{s}-f}{f_{s}+\left(f/\beta \right)}$$ (14)

where *v*_0_ is a constant value which is the shortening velocity of muscle when the *f* equals to 0. *f*_*s*_ is the stall force, the maximum force that single mysoin can bear. Myosin is forced to unbind when external force exceeds its stall force. *β* represents a dimensionless value that can modulate the force-velocity curve.

In addition, the PCM has been expanded to describe the slip-bond and catch-slip-bond properties of specific proteins (Erdmann et al., 2016; Chandrasekaran et al., 2019; Cortes et al., 2020). Due to the similar mechanisms, PCM can also be adopted to reproduce the myosin behaviors in smooth muscles. However, it should be noticed that the myosin ensembles *in vivo* interact with a more complicated environment. In this situation, the external force acting on the ensemble can result from the friction with sarcoplasm and interactions with other elastic proteins like the titin (Herzog, 2017). Therefore, the external force is not a constant value in the real muscle cell. Furthermore, since the PCM adopts a separated method to calculate the effective bound velocity, the result is mainly based on the stochastic property of the ensemble. Hence, the external forces from the environment or other proteins cannot be substituted in the force calculation in the PCM directly (Erdmann et al., 2013; Cortes et al., 2020). To overcome this problem, the force-velocity relation generated by the PCM should be considered as a reference. The myosin kinetics should be updated in each time step based on the varying external force and its corresponding effective velocity derived from the PCM. Then, a more accurate simulation can be achieved which comprehends the muscle activities well from a molecular perspective.

## 5 Conclusion

From the Hill’s muscle model, the generated muscular force can be separated into two parts: active and passive. The active part refers to the force generated by the muscle’s contractile mechanism in response to central nervous system activation. The passive component refers to the force that is generated due to the muscle’s material inherent properties. To validate the Hill-based muscle model from the molecular aspect, the catch-bond feature of the myosin-II protein should be considered. With this catch-bond property, external force can be generated in skeletal muscle through interactions between the myosin ensemble and F-actins. The parallel cluster model can quantitatively simulate the interactive behaviors of myosin. Significantly, the derived force-velocity relationship fits the Hill muscle model greatly, which can support that from a molecular perspective. With this confident molecular support, it is now possible for the controls researchers and engineers to develop the human-centered controllers for the functional electrical stimulation therapy which is a technique that generates the muscle tensile force through using the low-energy electrical pluses. The amount of force generated artificially can be calculated based on force-velocity relation of skeletal muscle. In addition, with this validation, it becomes potential that the musculoskeletal system can be modeled far more physiologically, forming the basis of the neuromechamics, which defines that the motion performed by the interaction among the neural, muscular, and skeletal system.
